# PReS-FINAL-1021: Further characterization of CD161+ regulatory t cells in health and disease

**DOI:** 10.1186/1546-0096-11-S2-P17

**Published:** 2013-12-05

**Authors:** CL Duurland, G Chain, RF O'Shaughnessy, AM Pesenacker, LR Wedderburn

**Affiliations:** 1 Rheumatology, London, UK; 2 Immunobiology, UCL Institute of Child Health, London, UK

## Introduction

Regulatory T cells (Treg) are crucial for maintaining immune homeostasis and mediating immune tolerance. Besides their immune regulatory function, Treg can produce pro-inflammatory cytokines such as interleukin (IL)-17 and interferon (IFN)-γ. Recently, our group has identified CD161 as a marker for pro-inflammatory FoxP3+ T cells which have classic Treg characteristics but also have effector T cell functions (Pesenacker *et al*., 2013). These data also demonstrated enhanced levels of CD161 expressing Treg within the inflamed synovial joints of Juvenile Idiopathic Arthritis (JIA) patients suggesting a role for CD161+ Treg in disease. Therefore, further characterization of this specific subset of Treg is required to unravel its function in health and disease.

## Objectives

The aim of this study was to define the phenotype of the recently identified CD161 expressing Treg population in healthy controls and JIA patients.

## Methods

CD161+ Treg within peripheral blood mononuclear cells (PBMC) of healthy controls (HC) (HC PBMC) and synovial fluid mononuclear cells (SFMC) of JIA patients (JIA SFMC) were analyzed by flow cytometry for the expression of Helios, programmed death (PD) 1, chemokine (CXC motif) receptor 3 (CXCR3), cytotoxic T lymphocyte-associated protein 4 (CTLA4), glucocorticoid-induced tumor necrosis factor receptor (GITR) and glycoprotein A repetitions predominant (GARP).

## Results

Clear phenotypic differences were observed between CD161+ and CD161- Treg. CD161+ Treg expressed lower levels of Helios compared to CD161- Treg in HC PBMC and JIA SFMC. In addition, CD161+ Treg showed an increased expression of PD1 compared to CD161- Treg within HC PBMC. Expression of PD1 was high on both CD161+ and CD161- Treg in JIA SFMC compared to HC PMBC. Furthermore, CD161+ Treg from HC PMBC expressed a slightly higher level of CXCR3 compared to their CD161- counterparts. Among JIA SFMC, the expression of CXCR3 was increased compared to the levels within HC PMBC. The percentage of CD161+ Treg expressing CTLA4 on the cell surface was increased compared to CD161- Treg in HC PBMC and JIA SFMC. Total CTLA4 expression was enhanced on both CD161+ and CD161- Treg in JIA SFMC. Expression of GITR by CD161+ Treg was increased compared to CD161- Treg among HC PBMC and JIA SFMC. Also, CD161+ Treg expressed higher levels of GARP compared to CD161- Treg among HC PBMC.

## Conclusion

The selected markers are all involved in mechanisms by which Treg exert their immune regulatory function. Therefore, the observed phenotypic differences between CD161+ and CD161- Treg may indicate differences in functional mechanisms used by these two different Treg populations in health and disease. Divergent functional capacity between CD161+ and CD161- Treg could explain the dual function of CD161 Treg in health and disease. These data might contribute towards development of new and/or optimized treatment strategies for JIA.

## Disclosure of interest

None declared.

